# Mastery Goals for Black American Dementia Caregivers

**DOI:** 10.1093/geroni/igab046.1994

**Published:** 2021-12-17

**Authors:** Kalisha Bonds Johnson, Fayron Epps, Glenna Brewster, Carolyn Clevenger, Gaea Daniel, Victoria Pak, Sudeshna Paul, Kenneth Hepburn

**Affiliations:** 1 Emory University, Atlanta, Georgia, United States; 2 Emory University, Stonecrest, Georgia, United States; 3 Emory University, Emory University, Georgia, United States

## Abstract

About 5.8 million older American adults live with Alzheimer’s disease and related dementias; Black American older adults’ prevalence is more than twice that of non-Hispanic white older adults. The Black American dementia caregiving experience can be pictured within the Black Family Social-Ecological Context Model, which provides a conceptual basis for examining social determinants of health at individual, family, community, and societal levels with careful consideration for how the intersecting identities of race, gender, and class of Black American caregivers influence the multiple dimensions of their caregiving experiences. Family dynamics, community setting, and healthcare systems have a potentially bidirectional influence on these caregivers, which is informed by the larger historical reality of systemic racism and general disenfranchisement. This paper outlines how Stress Process and Perceived Control frameworks offer ways for Black American dementia caregivers to achieve a sense of mastery within the complicated and fraught ecology within which their caregiving occurs. We propose a research and development agenda to create a program for enhancing a sense of mastery among Black American dementia caregivers. Two concepts in particular, “constraints” and “efficacy expectations,” provide ways to develop a systematic approach to developing successful coping strategies for the constraints perceived by individuals as they undertake and function in the caregiving role. The recognition of the complexity of the caregiving ecosystem and intersectionality of caregivers’ experience emphasize the importance of individualization: each caregiver’s experience of this ecosystem– and therefore each Black American caregiver’s way to mastery within it– will be uniquely shaped and experienced.

